# Proximity of Substantia Nigra Microstimulation to Putative GABAergic Neurons Predicts Modulation of Human Reinforcement Learning

**DOI:** 10.3389/fnhum.2017.00200

**Published:** 2017-05-09

**Authors:** Ashwin G. Ramayya, Isaac Pedisich, Deborah Levy, Anastasia Lyalenko, Paul Wanda, Daniel Rizzuto, Gordon H. Baltuch, Michael J. Kahana

**Affiliations:** ^1^Department of Neurosurgery, Perelman School of Medicine, University of PennsylvaniaPhiladelphia, PA, USA; ^2^Department of Psychology, University of PennsylvaniaPhiladelphia, PA, USA

**Keywords:** substantia nigra, human, dopamine, GABA, neuron, reinforcement learning, microstimulation, Parkinson's disease

## Abstract

Neuronal firing in the substantia nigra (SN) immediately following reward is thought to play a crucial role in human reinforcement learning. As in Ramayya et al. (2014a) we applied microstimulation in the SN of patients undergoing deep brain stimulation (DBS) for the treatment of Parkinson's disease as they engaged in a two-alternative reinforcement learning task. We obtained microelectrode recordings to assess the proximity of the electrode tip to putative dopaminergic and GABAergic SN neurons and applied stimulation to assess the functional importance of these neuronal populations for learning. We found that the proximity of SN microstimulation to putative GABAergic neurons predicted the degree of stimulation-related changes in learning. These results extend previous work by supporting a specific role for SN GABA firing in reinforcement learning. Stimulation near these neurons appears to dampen the reinforcing effect of rewarding stimuli.

## 1. Introduction

Thorndike's “Law of Effect” states that rewards strengthen associations between preceding stimuli and actions, resulting in reinforcement learning (Thorndike, 1932). Animal studies have shown that the phasic firing of substantia nigra (SN) neurons may represent a neural mechanism underlying reinforcement learning. SN dopamine (DA) neurons display phasic bursts that encode reward prediction error (RPE), a latent variable that tracks subsequent changes in associative strength (Sutton and Barto, 1990; Montague et al., 1996; Schultz et al., 1997; Bayer and Glimcher, 2005). They send prominent projections to dorsal striatal regions (Montague et al., 1996; Haber et al., 2000) that mediate action selection (Williams et al., 2006; Lau and Glimcher, 2008). Furthermore, DA release in the striatum has been shown to cause reinforcement of preceding actions and increased cortico-striatal synaptic strength (Reynolds et al., 2001).

Whereas animal studies have established a relation between SN neural firing and reinforcement learning, direct evidence from human studies is lacking. Patients undergoing deep brain stimulation (DBS) surgery for the treatment of Parkinson's Disease (PD) offers a rare opportunity to directly study the functional role of phasic SN activity during reinforcement learning (Jaggi et al., 2004; Zaghloul et al., 2009). Microstimulation, a technique that is widely used in animals to causally relate neural activity to behavior (Histed et al., 2009; Clark et al., 2011), is routinely applied as part of clinical protocol to aid in targeting of the DBS electrode. Patients are awake during this process to allow for detection of potential DBS-related adverse effects, and are able to perform cognitive tasks. In the only prior study relating microstimulation to human learning (Ramayya et al., 2014a), we showed that SN microstimulation near putative DA neurons impaired performance on a reinforcement learning task where rewards were contingent on stimuli, but unrelated to actions. Because of the experimental design used in this prior study, the observed stimulation-related decrease in performance could either signify impaired stimulus-reward learning, or a selective strengthening of action-reward associations that competed with stimulus-reward associations; the latter hypothesis was supported by further computational analyses (also, see de Berker and Rutledge, 2014).

In this study, we sought to clarify the role of phasic SN neural firing in human reinforcement learning. We applied SN microstimulation in eleven patients as they performed a reinforcement learning task with consistent stimulus-response mapping. In this task, stimulus-reward and action-reward associations were always correlated, and thus there was no confound between impaired learning and a selective strengthening of action-reward associations; improved performance suggests increased learning, whereas decreased performance suggests decreased learning. Because microstimulation has been shown to enhance the activity of neurons near the electrode tip (Histed et al., 2009), and because the human SN contains both DA and GABAergic neurons that represent functionally distinct populations (Ramayya et al., 2014b), we hypothesized that SN microstimulation would alter learning in a manner that was dependent on the properties of neurons near the electrode tip. Specifically, we expected stimulation-related improvements in learning when the electrode was positioned near putative DA neurons, but stimulation-related impairments in learning when the electrode was positioned near putative GABA neurons, that have been shown to exert inhibitory control over DA neurons (Tepper et al., 1995; Lobb et al., 2011; Henny et al., 2012; Pan et al., 2013).

## 2. Materials and methods

### 2.1. Subjects

Eleven patients undergoing Deep Brain stimulation (DBS) surgery for the treatment of Parkinson's Disease volunteered to take part in this study (6 male, 5 female, mean age = 63.8 years). Subjects provided their informed consent during pre-operative consultation and received no financial compensation for their participation. Per routine clinical protocol, Parkinson's medications were stopped on the night before surgery (12 h preoperatively); hence subjects engaged in the study while in an OFF state. The study was conducted in accordance with a University of Pennsylvania Institutional Review Board-approved protocol.

### 2.2. Intra-operative methods

**Figure 1:**
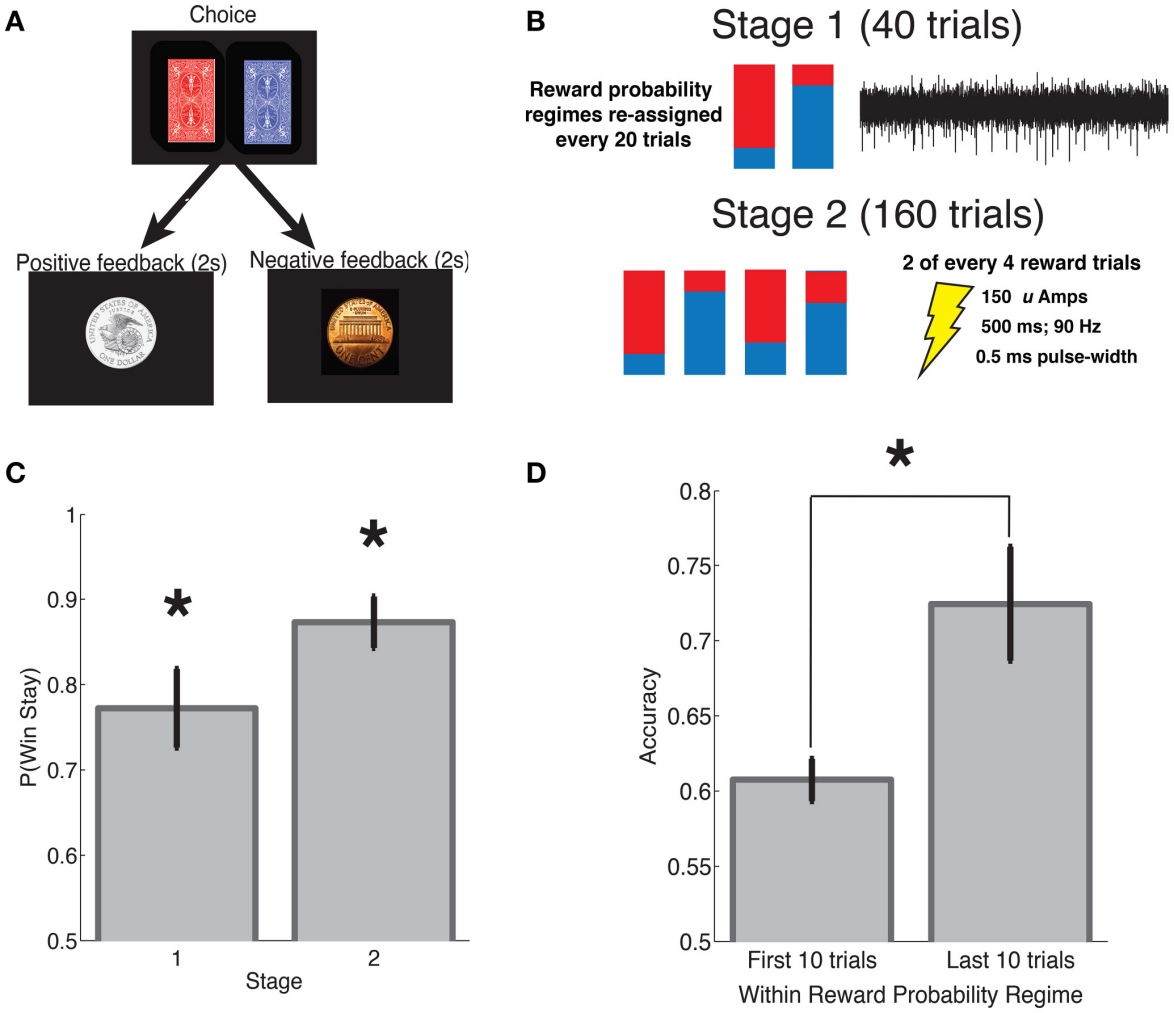
**Reinforcement learning task**. **(A)** Subjects performed a reinforcement learning task with consistent stimulus-response mapping. The visual stimuli presented during the choice and feedback interval are shown. Feedback was provided probabilistically in accordance with one of four reward probability regimes that were re-assigned every 20 trials. **(B)** Each subject's intra-operative session was divided into two stages. During stage 1 (40 trials), we obtained microelectrode recordings and the assigned reward probabilities were either 0.8:0.2 or 0.2:0:0.8 red:blue, whereas during stage 2 (160 trials) we applied SN microstimulation, and the assigned reward probabilities were one of the following: 0.8:0.2, 0.7:0.3, 0.3:0.7, or 0.2:0:0.8). See Section 2 for additional details. **(C)** Subjects demonstrated a greater win-stay than expected by chance during both stage 1 and 2. **(D)** Subjects made the high reward probability choice with greater frequency during the last 10 trials of a reward probability regime as compared to the first 10 trials. Error bars indicate standard error of mean (s.e.m) across subjects. ^*^indicates *p* < 0.001; see main text for statistics.

During surgery, intra-operative microelectrode recordings (obtained from a 1 μm diameter tungsten tip electrode advanced with a power-assisted microdrive) were used to identify the substantia nigra (SN) and the subthalamic nucleus (STN) as per routine clinical protocol (Jaggi et al., 2004) (Figure 1A). Electrical microstimulation is routinely applied through the microelectrode to aid in clinical mapping of SN and STN neurons, and was approved for use in this study by the University of Pennsylvania IRB. Once the microelectrode was positioned in the SN, we administered a two-alternative probability learning task through a laptop computer placed in front of the subject. Subjects viewed the computer screen through prism glasses placed over the stereotactic frame and expressed choices by pressing buttons on handheld controllers placed in each hand.

### 2.3. Reinforcement learning task

Subjects performed a two-alternative forced choice task with feedback. Each subject performed a single intra-operative session that consisted of two stages as described below. They also performed a pre-operative practice session that we did not include in our analyses. During each trial, subjects were presented with a pair of stimuli (red card deck and blue card deck), and asked to make a selection by pressing a button on one of two hand-held controllers (one in the left hand and one in the right hand). The red and blue card decks were presented simultaneously and arranged such that one deck was associated with a left button press, whereas the other deck was associated with a right button press. The arrangement of stimuli on the screen was randomly determined at the beginning of the experiment and remained fixed throughout.

Following each selection, subjects probabilistically received positive or negative feedback. Positive feedback was indicated by the appearance of a silver dollar accompanied by the audible ring of a cash register; negative feedback was indicated by the appearance of a copper penny accompanied by an error tone. The timing of each experiment was as follows: stimulus presentation and response time (variable), feedback presentation for 2 s and a 0–400 ms jitter between trials. Each experimental session consisted of 200 trials and was divided into two stages. During stage 1 (40 trials), we obtained microelectrode recordings from the SN, whereas during stage 2 (160 trials), we applied microstimulation following a subset of reward trials (see Section 2.4). To encourage subjects to attend to the rewards throughout the task, we employed a regime-switch model such that the reward probabilities associated with each of the card decks fluctuated throughout the experiment. In general, every 20 trials, the reward probabilities associated with the red and blue deck were assigned to one of several reward probability regimes. During stage 1, at trial 1 and trial 20, reward probabilities were assigned to one of two regimes, 0.8:0.2 or 0.2:0.8 (red:blue reward probability). During stage 2, every 20 trials, reward probabilities were assigned to one of four regimes (red:blue): 0.8:0.2, 0.7:0.3, 0.3:0.7, and 0.2:0.8. Before beginning the task, patients were shown an introductory video describing the task. Patients also participated in a pre-operative practice session of the task prior to the intra-operative session. On average, subjects had a response time of 1.80 ± 1.00 s (mean ± s.d.) per trial, and the intra-operative experiment lasted 15.57 ± 2.45 m (mean ± std).

As compared to the task used in Ramayya et al. (2014a), the current task included the following changes. First, only one set of stimuli (a red and blue card deck) were presented throughout the experiment instead of multiple stimulus pairs. Second, the stimuli were presented in the same arrangement on the screen from trial to trial such that there was consistent stimulus-response mapping. In other words, for a given experimental session, the red card was always presented on the left and the blue card was always presented on the right, associated with left and right button presses, respectively. Third, because only one set of stimuli were presented throughout the experimental session, we employed a regime-switch design to encourage learning throughout the task as described above.

### 2.4. Stimulation parameters

We applied microstimulation immediately following feedback on approximately half of the reward trials during stage 2 (the latter 160 trials) of each intra-operative session. Specifically, we applied stimulation following 2 of every 4 reward trials that were pseudorandomly determined at the beginning of each experiment. Stimulation was provided through the microelectrode immediately following feedback presentation during the learning task using an FHC Pulsar 6b microstimulator using the following parameters: bi-phasic, cathode phase-lead pulses at 90 Hz, lasting 500 ms at an amplitude of 150 Amps and a pulse width of 500 μs. These stimulation parameters were used in our previous SN microstimulation study (Ramayya et al., 2014a), and similar parameters have induced learning in the rodent SN (Reynolds et al., 2001) and the non-human primate VTA (Grattan et al., 2011). An LED on the front chasse of the stimulator indicated the onset of stimulation, however, this was not visible to the patient as they performed the task. There was no sound associated with stimulation. Thus, stimulation trials were not signaled to subjects in any manner. None of the subjects reported a perceptual change following the application of microstimulation.

### 2.5. Extracting spiking activity from microelectrode recordings

We obtained microelectrode recordings during the first 40 trials of each intra-operative session prior to applying microstimulation during the experiment. Because these recordings were of a relatively short duration (≈ 5 min), their main purpose was to aid in interpretation of the stimulation results, rather than to characterize the functional properties of human SN neuronal activity (Zaghloul et al., 2009; Ramayya et al., 2014b). To assess whether stimulation-related behavioral changes were related to the properties of neurons near the electrode tip, we extracted multi-unit activity following methods previously described (Ramayya et al., 2014a,b).

Briefly, we extracted neuronal activity from each microelectrode recording using the WaveClus software package (Quiroga et al., 2005) after band-pass filtering the signal and manually removing periods of motion artifact. We identified spike events as positive or negative deflections in the voltage trace that crossed a threshold that was manually defined for each recording (≈ 3.5 S.D.). We used both positive and negative voltage fluctuations to identify units, rather than only negative deflections as in our previous microstimulation study (Ramayya et al., 2014a), because our recent electrophysiological study demonstrated that positive voltage fluctuations also contain task-related unit activity (Ramayya et al., 2014b). Spikes were subsequently clustered into units based on the first three principal components of the waveform and noise clusters from motion artifact or power line contamination were manually invalidated. We considered positive and negative deflections in the voltage signal to be independent units, but otherwise combined spiking activity on a given channel into multi-unit activity. We identified between 1 and 2 multi-units on each recording channel, except for one subject (#8) where we could not distinguish spiking activity from noise contamination (Table 1). When 2 multi-units were recorded from a single subject, we considered baseline firing rate to be the average baseline firing rate of the two contributing units to account for the artificial elevation in firing rate that results from combining units.

**Table 1:** **Summary of participant data**.

**Subject**	**Age**	**Gender**	**Accuracy**	**Win-stay no-stim**	**△win-stay**	**Mean spike rate**	**Mean waveform duration**
1	46	M	0.61	0.76	+0.03	20.0	0.74
2	62	F	0.60	0.84	+0.02	5.19	1.04
3	50	M	0.84	1	−0.02	12.8	0.44
4	68	F	0.87	1	0	7.15	1.04
5	68	M	0.74	1	0	14.7	0.92
6	75	M	0.63	0.85	−0.07	48.2	0.48
7	60	M	0.57	0.73	−0.08	20.4	0.44
8	66	F	0.60	0.81	−0.01	−	−
9	69	F	0.72	0.93	−0.04	17.4	0.40
10	66	M	0.68	0.89	−0.09	33.0	0.40
11	72	F	0.70	0.94	−0.02	10.9	0.16

*Columns 4–6 describe behavioral changes during stage 2. Columns 7–8 describe properties of multi-unit activity recorded during stage 1. “–” indicates missing data. We did not identify spiking activity from subject #8*.

### 2.6. Identifying putative dopaminergic and GABAergic neurons

To study effect of microstimulation on SN DA and GABA neurons, we sought to assess the location of the microelectrode relative to each of these neural populations. Because DA and GABA neurons are locally clustered but largely interspersed in the SN (Poirier et al., 1983), putative DA and GABA neurons are typically identified based on physiological and functional properties of neurons, rather than their relative location within the SN (Fiorillo et al., 2013). Because of the limited intra-operative time, and technical challenges in simultaneously recording neural activity and applying microstimulation, we obtained neural recordings for a short duration (stage 1, ≈ 5 min) from a particular site in the SN prior to applying stimulation at that site. We sought to leverage findings from prior dedicated electrophysiology studies in animals (Ungless and Grace, 2012) and humans (Ramayya et al., 2014b) infer proximity of the microelectrode to these respective neuron types.

Previous studies which have combined electrophysiological recordings with pharmacological manipulations (Schultz and Romo, 1987) or histochemical techniques (Henny et al., 2012) have shown that DA neurons exhibit slow firing rates and broad waveforms, whereas GABA neurons display fast firing rates and narrow waveforms (Ungless and Grace, 2012). In a previous study, we showed that SN single-units that demonstrated firing rates slower than 15 Hz and waveform durations >0.8 ms demonstrated post-feedback responses consistent with DA neurons, whereas units that demonstrated high spike rates (>15 Hz) and narrow waveforms (<0.8 ms) demonstrated post-feedback responses consistent with GABAergic neurons (Ramayya et al., 2014b), a finding consistent with prior non-human primate studies (Matsumoto and Hikosaka, 2009).

In the current study, because of limited recording time and a limited number of subjects, we did not seek to identify distinct DA and GABA units to study as separate groups. Instead, we sought to extract physiological parameters of multi-unit activity that could serve as biomarkers of putative DA or GABA neural populations near the microelectrode. We extracted three physiological features from each unit as indicators of putative DA and GABAergic activity (Ungless and Grace, 2012; Ramayya et al., 2014a,b): mean spike rate, waveform duration (computed as peak-to-trough duration), and phasic post-reward activity (the difference between the average spike rate during 0–500 ms post-reward interval, and that during the −250–0 and 500–750 ms intervals). We sought to assess whether these physiological parameters could predict the effect of microstimulation on behavior. We used this approach in our previous microstimulation study (Ramayya et al., 2014a) to uncover a relation between the effect of stimulation and the properties of neurons recorded near the electrode tip.

### 2.7. Statistical analyses

Unless otherwise noted, we performed across subject analyses whereby each subject contributed one observation to each statistical test. We used Student *t*-tests to compare mean value of continuous distributions, and Pearson's correlation *r* when studying the linear dependence between two variables. We considered a *p* < 0.05 to be statistically significant.

## 3. Results

We applied intra-operative microstimulation in the SN of eleven patients undergoing DBS for the treatment of PD as they performed a reinforcement learning task (Table 1). Subjects selected between a red and blue card deck by pressing buttons on hand-held controllers and subsequently received positive or negative feedback (Figure 1A). The reward probabilities associated with each card deck stochastically fluctuated throughout the intra-operative session to encourage learning (Figure 1B, see Section 2).

Subjects demonstrated clear evidence of learning on the task. Both during stage 1 and stage 2, subjects showed an increased probability of repeating the same action after receiving positive feedback [“win-stay,” 0.5 expected by chance; *t*_(10)_ > 5.8, *p*'s < 0.001, Figure 1C]. Subjects also showed an increased probability of making a high reward probability choice (“accuracy”) during the last 10 trials of a particular reward probability regime, as compared to the first 10 trials after a regime switch [*t*_(10)_ = 4.35, *p* = 0.001, Figure 1D].

**Figure 2:**
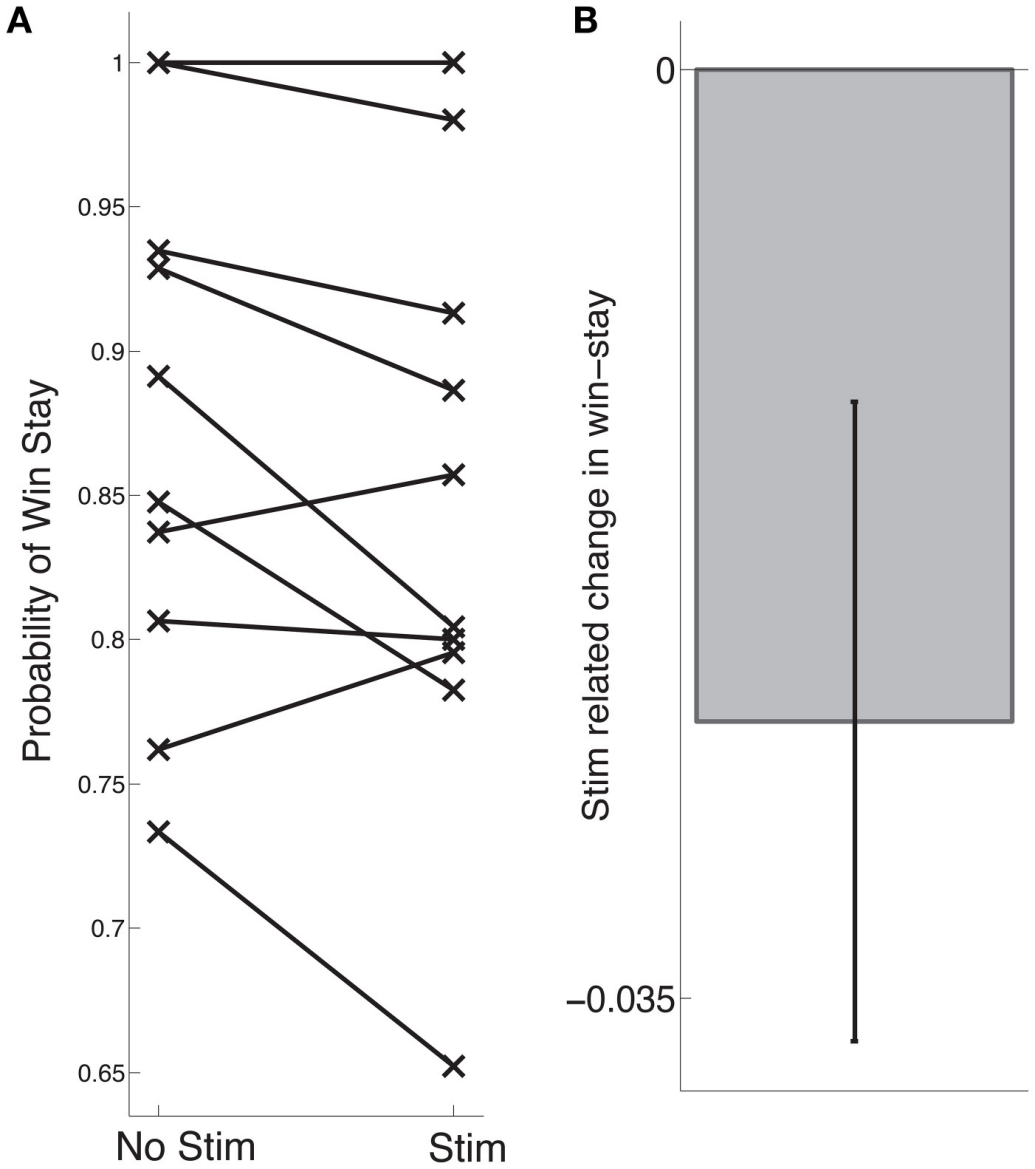
**Stimulation-related change in learning**. **(A)** Each subjects' probability of win stay during stage 2 is indicated by an “x,” following control trials on the left and following stimulation trials on the right. **(B)** Across subjects, we observed a trend toward an stimulation-related decrease in learning (*p* = 0.068). Error bars indicate standard error of mean (s.e.m) across subjects; see main text for statistics.

To assess the importance of SN neuronal activity for learning, we applied SN microstimulation following approximately half the reward trials during stage 2 of each subject's intra-operative session. To assess whether SN stimulation had an effect on learning, we compared subjects' win-stay probabilities following reward trials that were accompanied by stimulation (“stim trials”) and stage 2 reward trials during which stimulation was not applied (“control trials”). Across 11 subjects, we observed a trend toward decreased win-stay following stimulation trials compared to control trials [*t*_(10)_ = 2.03, *p* = 0.068, Figure 2].

**Figure 3:**
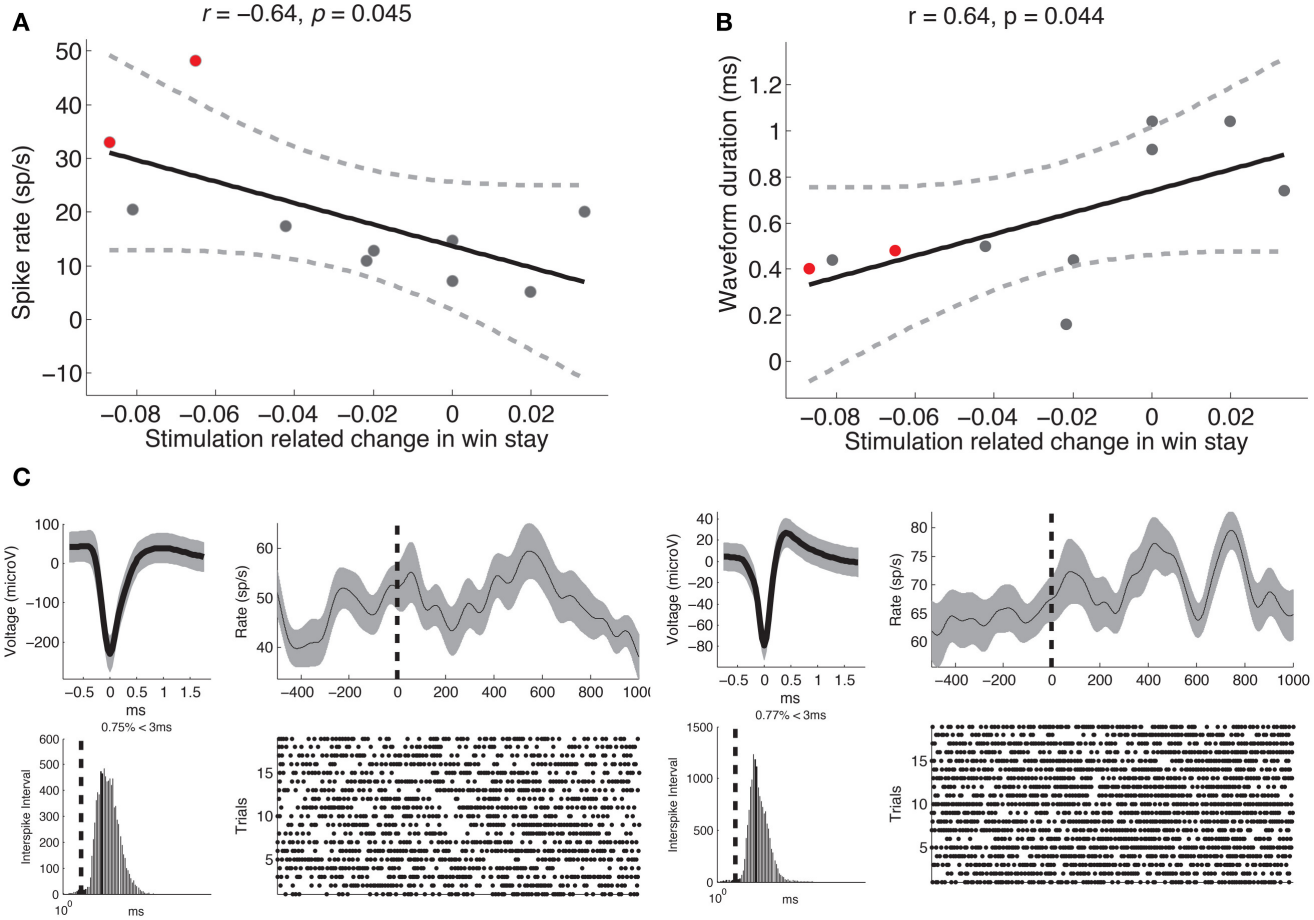
**Stimulation-related changes in learning are related to recorded neural activity**. **(A)** Stimulation-related changes in learning during stage 2 were negatively correlated with mean spike rate of units recorded on that electrode during stage 1 (Pearson's *r* = −0.64, *p* = 0.045). **(B)** Same as **(A)** but demonstrating a positive correlation between stimulation-related changes in learning and mean waveform duration (Pearson's *r* = 0.64, *p* = 0.044). Each dot represents a subject, the solid black line is the regression slope, and the dashed lines represent 95% confidence intervals. **(C)** Neural recordings of multi-unit activity observed from two subjects (shown in red in **A,B**). For each unit, we show the average waveform (top left, gray shading marks the standard deviation), the inter-spike interval (bottom left, dashed line marks 3 ms), the average post-reward firing response (top right, smoothed with a Gaussian kernel of half-width = 75 ms; gray shading indicates s.e.m), and the spike raster following reward trials. Dashed black line indicates reward onset.

Our main hypothesis was that stimulation-related changes in learning would vary based on the functional properties of neurons near the electrode tip. To assess whether this was the case, we extracted various physiological parameters from neural activity recorded during stage 1 of each subject's intra-operative session (see Section 2). We assessed whether there was a correlation between stimulation-related changes in learning and mean spike rate of units recorded on each channel, and observed a significant negative correlation such that the greatest impairments in learning were observed when the electrode was positioned near neurons with relatively high spike rates (*r* = −0.64, *p* = 0.045, Figure 3A). Based on the the established finding that high spike rates and narrow waveforms are properties of GABAergic neurons (Ungless and Grace, 2012), we also assessed for a correlation between stimulation-related changes in learning and mean waveform duration. We observed a positive correlation between stimulation-related changes in learning and waveform duration, such that the strongest impairments occurred near neurons with narrow waveforms (*r* = 0.64, *p* = 0.044, Figure 3B). We did not observe a significant relation between stimulation-related changes in learning and phasic post-reward changes in activity (*p* > 0.5), and generally did not observe post-reward phasic changes in activity (*z*-score range: −0.1:0.36). Two example neurons are shown in Figure 3C.

## 4. Discussion

We applied microstimulation in SN of patients undergoing DBS for the treatment of PD as they performed a reinforcement learning task. We found that microstimulation applied during the 500-ms post-reward interval impaired learning. These results demonstrate a causal relation between post-reward SN firing and human reinforcement learning as microstimulation is known to acutely enhance local neural firing (Histed et al., 2009). We hypothesized that the effect of SN microstimulation on learning would vary based on their relative proximity to dopaminergic (DA) neurons that guide reinforcement learning (Glimcher, 2011) or GABAergic neurons that exert inhibitory control on DA neurons (Damier et al., 1999a; Lobb et al., 2011; Ramayya et al., 2014b). As hypothesized, we observed the largest stimulation-related impairments in learning when the electrode was positioned near neurons with relatively high firing rates and narrow waveforms, properties characteristic of GABA neurons (Joshua et al., 2009; Matsumoto and Hikosaka, 2009; Ungless and Grace, 2012). Thus, our results suggest that microstimulation near GABA neurons impairs reinforcement learning.

This finding provides direct evidence relating phasic SN neural firing to human reinforcement learning. It goes beyond animal electrophysiology studies that may not generalize to human learning because they typically involve long periods of intense training. It also goes beyond prior human studies of reinforcement learning; functional neuroimaging studies cannot test a causal role for SN neural activity (Montgomery et al., 2009), and pharmacological manipulations of DA in patients with PD (Frank et al., 2004; Rutledge et al., 2009) cannot distinguish phasic neural activity from tonic changes in DA throughout the brain (Niv et al., 2007). Ramayya et al. (2014a) also showed a stimulation-related decrease in performance. However, because rewards in that study were contingent on stimuli, but independent of actions, the observed stimulation-related decrease in performance could either be attributed to an impairment of learning or a selective strengthening of action-reward associations that competed with stimulus-reward learning. Our current study overcame this limitation by using an experimental design with consistent stimulus-response mapping, such that stimulus-reward and action-reward associations were always correlated. Thus, our finding of a stimulation-related impairment in performance suggests decreased learning.

In Ramayya et al. (2014a), stimulation-related decreases in performance were correlated with an increased propensity to repeat the same action following reward, particularly when the electrode was positioned near putative DA neurons, suggesting that microstimulation near SN DA neurons enhanced action-reward learning. The current finding that stimulation near putative GABA neurons produced impairments in reinforcement suggests opposing roles of DA and GABA neurons during reinforcement learning. Specifically, if phasic bursts of SN DA neurons encode reward prediction errors that result in subsequent learning (Glimcher, 2011), and SN GABA neurons provide inhibitory inputs to local DA neurons (Tepper et al., 1995; Luscher and Ungless, 2006; Lobb et al., 2011; Henny et al., 2012; Pan et al., 2013), then one would observe enhanced learning when stimulating DA neurons (Ramayya et al., 2014a), but impaired reinforcement learning following microstimulation of SN GABA neurons. This explanation is also supported by our observation of opposing post-reward firing responses from putative DA and GABA neurons in the human (Ramayya et al., 2014b).

It is difficult to interpret whether the observed changes reinforcement learning were related to changes in stimulus-reward and/or action-reward learning because these forms of learning were perfectly correlated in the current experimental design. That we did not observe robust stimulation-related changes in learning near putative DA sites is difficult to interpret when considering our previous finding that microstimulation near putative DA neurons enhances action-reward learning (de Berker and Rutledge, 2014; Ramayya et al., 2014a). It is possible that we did not sample from a functional population of DA neurons in this study, as suggested by the absence of phasic post-reward bursts in activity from putative DA neurons in this study, unlike our previous studies (Ramayya et al., 2014a,b). Alternatively, it is possible that stimulation near SN DA neurons has a specific effect on action-reward learning that was not evident in this study because it was masked by simultaneous stimulus-reward learning.

An alternative explanation for how microstimulation of SN GABA neurons might have resulted in impaired learning is that stimulation may have caused a behavioral change during the post-reward interval that impaired subjects' learning during those trials. Several studies have linked the firing of SN GABA neurons in the *pars reticulata* subregion (that contains the majority of SN GABA neurons; Nair-Roberts et al., 2008) to regulation of downstream movement and saccade-generating structures (e.g., superior colliculus; Carpenter et al., 1976; DeLong et al., 1983; Hikosaka and Wurtz, 1983). If microstimulation of SN GABA neurons suppressed orienting saccades that likely occurred in response to the presentation of salient reward stimuli (in this case, a silver dollar and the sound of cash register; Hikosaka and Wurtz, 1983), then reward stimuli presented during stimulation trials might be associated with diminished salience and result in reduced learning. However, this is unlikely to be the case because non-human primate studies have shown that SN microstimulation has a limited influence on visually-guided saccades (Mahamed et al., 2011).

We note several limitations to our study. First, we are unable to provide direct histochemical evidence that electrophysiological parameters (spike rate and waveform duration) indicate distinct neuronal populations, however, a large body of evidence from animal studies suggest that these electrophysiological criteria may be used to identify distinct midbrain neuronal populations (Ungless and Grace, 2012). Second, we did not observe stimulation-related changes in learning near putative DA neurons in this study, whereas we observed such changes in our previous microstimulation study (Ramayya et al., 2014a). This likely reflects reduced sampling of DA neurons during this experiment, which is consistent with the fact that we did not observe post-reward bursts of activity in this study (a marker of DA activity), in contrast to Ramayya et al. (2014a). Finally, the population we studied—patients undergoing DBS surgery for PD—is known to have degeneration of DA neurons in SN. Even though this poses the challenge of interpreting findings concerning the functional role of SN neurons in patients who have degenerative disease, histological studies in PD patients (Damier et al., 1999b), and electrophysiological studies in rat models of PD (Hollerman and Grace, 1990; Zigmond et al., 1990), and humans (Zaghloul et al., 2009; Ramayya et al., 2014b) indicate that a significant population of viable neurons remain in the parkinsonian SN. Taken together with the clear evidence of learning that subjects demonstrated during the task, we suggest that the neural processes we describe reflect the subpopulation of healthy neurons that remain in the SN.

## 5. Conclusion

We demonstrate a specific role for SN GABAergic neural activity in human reinforcement learning. We found that the proximity of SN microstimulation near putative GABA neurons predicted impairments in learning, possibly related to local inhibition of phasic DA bursts. These results raise the possibility that SN microstimulation may allow for bi-directional control of reinforcement learning in pathological conditions (e.g., stimulation of GABA neurons to reduce learning during addiction, and stimulation of DA neurons enhance learning during stroke recovery). To further evaluate this possibility, future studies must improve intra-operative targeting of DA and GABA neurons and clarify the mechanisms by which SN microstimulation alters learning.

## Ethics statement

This study was carried out in accordance with the recommendations of University of Pennsylvania Institutional Review Board with written informed consent from all subjects. All subjects gave written informed consent in accordance with the Declaration of Helsinki. The protocol was approved by the University of Pennsylvania Institutional Review Board.

## Author contributions

AR, GB, IP, and MK designed research; AR, DL, AL, PW, and GB performed research; IP and AR analyzed the data; AR and MK wrote the paper. All authors contributed to the intellectual content of the research and provided final approval on the manuscript.

### Conflict of interest statement

The authors declare that the research was conducted in the absence of any commercial or financial relationships that could be construed as a potential conflict of interest.
